# Protective Effects of Trehalose on the Corneal Epithelial Cells

**DOI:** 10.1155/2014/717835

**Published:** 2014-06-18

**Authors:** Pasquale Aragona, Pietro Colosi, Laura Rania, Francesca Colosi, Antonina Pisani, Domenico Puzzolo, Antonio Micali

**Affiliations:** ^1^Department of Experimental Medical-Surgical Sciences, Regional Referral Center for the Ocular Surface Diseases, University of Messina, Via C. Valeria 1, 98125 Messina, Italy; ^2^Department of Social and Environmental Health, University of Messina, Via C. Valeria 1, 98125 Messina, Italy; ^3^Department of Biomedical Sciences and Morphofunctional Imaging, Section of Histology and Embryology, University of Messina, Via C. Valeria 1, 98125 Messina, Italy

## Abstract

*Purpose.* Aim of the present work was to evaluate the effects of the trehalose on the corneal epithelium undergoing alcohol delamination. *Methods.* Twelve patients undergoing laser subepithelial keratomileusis (LASEK) were consecutively included in the study. The right eyes were pretreated with 3% trehalose eye drops, whilst left eyes were used as control. Epithelial specimens were processed for cells vitality assessment, apoptosis, and light and transmission electron microscopy; a morphometric analysis was performed in both groups. *Results.* In both trehalose-untreated eyes (TUE) and trehalose-treated eyes (TTE), the percentage of vital cells was similar and no apoptotic cells were observed. In TUE, the corneal epithelium showed superficial cells with reduced microfolds, wing cells with vesicles and dilated intercellular spaces, and dark basal cells with vesicles and wide clefts. In TTE, superficial and wing cells were better preserved, and basal cells were generally clear with intracytoplasmatic vesicles. The morphometric analysis showed statistically significant differences between the two groups: the TTE epithelial height was higher, the basal cells showed larger area and clearer cytoplasm. The distribution of desmosomes and hemidesmosomes was significantly different between the groups. *Conclusions.* Trehalose administration better preserved morphological and morphometric features of alcohol-treated corneal epithelium, when compared to controls.

## 1. Introduction

Laser subepithelial keratomileusis (LASEK) is a surgical technique carried out on patients who undergo photorefractive keratectomy (PRK) for low myopia, with thin cornea or with professions or lifestyles that expose them to trauma, so contraindicating the laser in situ keratomileusis (LASIK) [1]. The procedure consists of a chemical reduction of the epithelial adhesion to Bowman's layer by the application of a dilute solution of ethanol on the corneal surface [1].

Despite the large number of works either in laboratory animals [2–4] or in humans, both in normal [1, 5–10] and in pathological eyes [11, 12], the effects of alcohol on the corneal epithelium are still controversial. In fact, no significant changes [1, 4, 6] or minimal adverse effects [2] to well-evident damages [3, 5, 7–9, 12] have been described. All these changes were related to the action of ethanol [12]. It acts by removing water and destabilizing either the protein hydrophobic bonds, thus unfolding the tertiary protein structure, or the hydrogen bonds in hydrophilic areas, resulting in protein denaturation [13]. Furthermore, alcohol penetrates the tissues and substitutes inter- and intracellular water: consequently shrinkage and hardening of tissues can be observed [14].

In order to prevent the morphological changes induced by alcohol on the corneal surface, the protective action of trehalose was considered. Trehalose is a nonreducing disaccharide of glucose, naturally produced, and accumulated in many living organisms, but not in mammals [15]. It was identified as a key response element needed to protect the cells against a great number of environmental stresses, such as desiccation, dehydration, cold, heat, and oxidation [13]. Among these functions, the protection against desiccation was widely studied in ophthalmic research, as exogenous trehalose protects corneal epithelial cells from experimental drying [16] and was shown to be effective in the treatment of moderate to severe human dry eye [17]. Furthermore, during desiccation in vivo, it was also demonstrated that trehalose could effectively suppress apoptotic cell death on the ocular surface [18].

Aim of the present work was to compare the structure and the ultrastructure of the corneal epithelium in patients undergoing alcohol delamination with and without trehalose pretreatment.

## 2. Materials and Methods

### 2.1. Study Design

This is an experimental, controlled study on a model of corneal epithelial alcohol delamination currently used in some refractive surgery procedures. It was carried at the Regional Referral Center for the Ocular Surface Diseases of the Department of Experimental Medical-Surgical Sciences of the University Hospital of Messina, Messina, Italy. Ethics approval was granted by the Institutional Review Board of the Department of Experimental Medical-Surgical Sciences of the University Hospital of Messina, Messina, Italy, and the study was conducted in concordance with the tenets of the Declaration of Helsinki. Informed consent was obtained from all the participants, after explanation of the nature and the possible consequences of the study.

### 2.2. Patients Population

The epithelial specimens were obtained from 24 eyes (12 patients; 7 male and 5 female; mean age 26.3 ± 4.2 years), with a refractive error of −4 ± 2.8 diopters, undergoing PRK. Inclusion criteria were subjects eligible for refractive surgical procedure with myopia in both eyes below 7 diopters, willing to participate to the study and to adhere to the study protocol, and who signed the informed consent. Exclusion criteria were systemic or ocular diseases contraindicating the refractive surgical procedure or subjects who were not willing to adhere to the study protocol.

### 2.3. Treatment Protocol

Before the surgical procedure, the right eyes of the patients were treated as follows: a drop of a solution based on 3% trehalose (Thealoz, Thea Farma, Milano, Italy), followed, after 5 min, by the instillation of a drop of 0.4% oxybuprocaine hydrochloride (Novesina, Novartis Farma, Origgio VA, Italy). This procedure was performed every 15′ for the hour before the surgical procedure, so that a total of 5 drops of trehalose were instilled within the hour. The left eyes were treated with only 0.4% oxybuprocaine hydrochloride instilled five times for the hour before the surgical procedure. During the surgical treatment, the epithelial specimens were obtained as follows: demarcation of a central corneal area with a 9.0 mm diameter cone (J2907, Janach, Como, Italy); filling of the cone with a 20% solution of ethyl alcohol in distilled water for 25 sec; emptying of the cone with a Merocel sponge (Medtronics Merocel, Mystic, CT, USA); washing with a solution 1 : 1 of balanced salt solution; and distilled water and successive drying with a Merocel sponge. The epithelial flaps were obtained by gently detaching the alcoholized epithelium with a blunt spatula and divided in three specimens for the morphological assessment.

### 2.4. Epithelial Cells Vitality Evaluation

Epithelial cells vitality was evaluated with the trypan blue dye test [6]. One specimen was incubated for 2′ in 0.1% trypan blue in phosphate-buffered saline (PBS) at 37°C. It was then washed several times in warm PBS and incubated in Dulbecco's modified Eagle culture medium (Sigma-Aldrich Srl, Milano, Italy) containing 10% of fetal calf serum for 30′ at 37°C. After a further wash in warm PBS, the specimen was flat mounted on a slide and photographed with a Zeiss Primo Star LM. Vital cells appeared with unstained nuclei and cytoplasm, while dead cells were blue.

### 2.5. Apoptosis Assay

One specimen was fixed in 10% formaldehyde in 0.2 M PBS, dehydrated in graded ethanol, cleared in xylene, and embedded in paraffin (Paraplast, SPI Supplies, West Chester, PA, USA). Paraffin blocks were placed in a rotary microtome (RM2125 RT, Leica Instruments, Nußloch, Germany), and 5 *μ*m sections, cut with disposable metal blades (S35, Feather Safety Razor Co, Osaka, Japan), were cleared with xylene and rehydrated in graded ethanol. An apoptosis detection kit (Chemicon International, ApopTag Plus Peroxidase In Situ Apoptosis Detection Kit, Temecula, CA, USA) was used [19]. The sections were subjected to partial digestion with proteinase K (20 *μ*g/mL) and washed in PBS. Endogenous peroxidase activity was blocked with 3% H_2_O_2_ in PBS. Thereafter, the sections were incubated in an equilibration buffer and then with the TUNEL reaction mixture in a humidified chamber at 37°C for 60 min. They were incubated in a stop/wash buffer at room temperature and washed with PBS. The anti-digoxigenin conjugate was applied and incubation in a humidified chamber was performed for 30 min at room temperature. The 3-3′-diaminobenzidine (DAB) was then added for 10 min, and the slides were counterstained with Mayer's hematoxylin. The sections were washed in ethanol and xylene, mounted in Permount (Fisher Scientific, Fair Lawn, NJ, USA), and photographed with a Zeiss Primo Star LM.

### 2.6. Light and Transmission Electron Microscopy

One specimen was immediately fixed in 2.5% glutaraldehyde 0.2 M in Sørensen's phosphate buffer (pH 7.2) at +4°C for 4 h, washed with 0.2 M Sørensen's phosphate buffer (pH 7.2), and postfixed in 1% osmium tetroxide (OsO_4_) in 0.2 M Sørensen's phosphate buffer (pH 7.2) at +4°C for 1 h. The specimen was dehydrated in graded ethanol and acetone and flatembedded in embedding agent (Durcupan ACM Fluka, Sigma-Aldrich, St. Louis, MO, USA) for a better orientation. Semithin sections (1 *μ*m) were cut with a LKB Ultrotome V ultramicrotome, stained with an aqueous solution of 1% toluidine blue in 1% borax and 1% pironine, and viewed and photographed with a Zeiss Primo Star light microscope (LM). From the same specimens used for LM, ultrathin sections of gold-silver interference color were cut with a diamond knife on a LKB Ultrotome V ultramicrotome, collected on uncoated 200–300 mesh copper grids, and stained with methanolic uranyl acetate and lead citrate. Micrographs were taken with a Philips CM-10 transmission electron microscope (TEM) at 80 kV.

### 2.7. Morphometric Analysis

A morphometric analysis of the corneal epithelium in both groups of eyes was carried out on LM micrographs, considering the percentage of vital cells, the number of epithelial layers, the epithelial thickness, the area of the basal cells, and the optical density of their cytoplasm. On TEM images, the number of desmosomes (De) of superficial, wing and basal cells and hemidesmosomes (Hd) of the basal cells were evaluated.

The percentage of vital cells was counted on 5 micrographs/eye obtained from 5 different, nonoverlapping, randomly chosen microscopic fields. For each field, a square of 150 × 150 *μ*m was drawn with the Adobe Photoshop 8.0.1 software and the cells included in the square were counted, including among the cells touching the square border only those from two sides.

LM data were obtained from 5 semithin sections/eye (total of 60 sections/group), collecting 1 semithin section every 100; all micrographs were obtained at the same magnification of 450x with a Zeiss Primo Star LM and processed with a Macintosh MacBook, using the Adobe Photoshop 8.0.1 software. All images were converted to black and white and brought to the final magnification of 1 000x for the morphometric analysis. To avoid possible bias from oblique sectioning, which would artificially increase the measured thickness, the thinnest portion of the epithelium was measured in all the images. On the selected images, a straight line perpendicular to the epithelium was traced. The number of epithelial layers was counted along the line. The overall epithelial thickness was expressed in *μ*m and calculated according to the following formula for the conversion from pixels: [(length in pixels) × (10 000 *μ*m/cm)]/[(resolution in pixels/cm) × (magnification)]. As to the basal cells area and their cytoplasmatic density, 10 microscopic fields of 100 *μ*m each were randomly chosen for every group and analyzed with the public domain ImageJ software (ImageJ, http://rsb.info.nih.gov/ij/). For the measurements, only basal cells with well evident nuclei were chosen: the area was expressed in *μ*m^2^ and the cytoplasmatic density was calculated (function: analyze > measure) in optical units (OU) within the levels from 0 (black) to 255 (white).

TEM morphometric analysis was performed on negative films (Kodak 4489) obtained from 5 ultrathin sections/eye at the same voltage, exposure time, and magnification. The negative films were acquired (ratio 1 : 1) with an Epson Perfection 4180 scanner, processed with a Macintosh MacBook, using the Adobe Photoshop 8.0.1 software, and converted into positive images at the same final magnification of 18 000x. The absolute number of De was calculated along the interface between adjacent superficial, wing and basal cells by counting all De observed in 100 standard fields of 10 *μ*m, each including the cellular membranes of two adjacent cells. The absolute number of Hd was calculated along the interface between basal cells and basement membrane, by counting all Hd observed in 100 standard fields of 10 *μ*m, each including a single cell.

Two experienced histologists, masked about the origin of the specimens, performed all the morphological and morphometric evaluations. All data were expressed in *μ*m for linear values, in *μ*m^2^ for cellular area, and in OU for optical density between 0 (black) and 255 (white).

### 2.8. Statistical Analysis

Primary efficacy variables were the epithelial thickness, the number of De, the area of the basal cells, and the optical density of the basal cells cytoplasm. Secondary parameters were the number of layers and the number of Hd. For the analysis of the results, the Student's *t*-test for unpaired data was used. A *P* value of ≤0.05 was considered statistically significant.

## 3. Results

### 3.1. Epithelial Cells Vitality Evaluation and Apoptosis Assay

**Figure 1:**
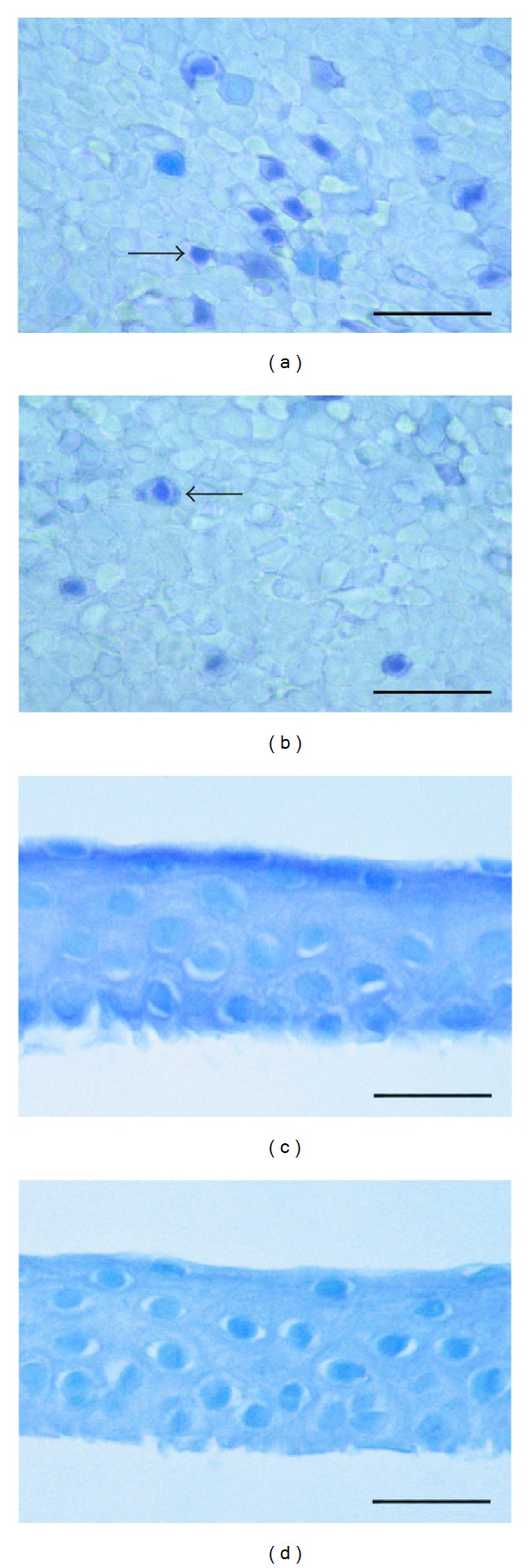
(a, b) Light micrographs of trypan blue dye test for epithelial cells vitality. In both TUE (a) and TTE (b) positive, dead cells (arrow) can be observed. Scale bar: 80 *μ*m. (c, d) Light micrographs of TUNEL reaction of corneal epithelium. In both TUE (c) and TTE (d) no positive cells can be observed. Scale bar: 30 *μ*m.

The trypan blue dye test demonstrated that trehalose-untreated eyes (TUE) (Figure 1(a)) showed a higher amount of not vital cells stained in blue with the dye than trehalose-treated eyes (TTE) (Figure 1(b)) (11.8 ± 0.8% versus 14.4 ± 0.9, resp., *P* < 0.001).

The apoptosis assay in both TUE (Figure 1(c)) and TTE (Figure 1(d)) failed to demonstrate positive cells.

### 3.2. Structural and Ultrastructural Data

**Figure 2:**
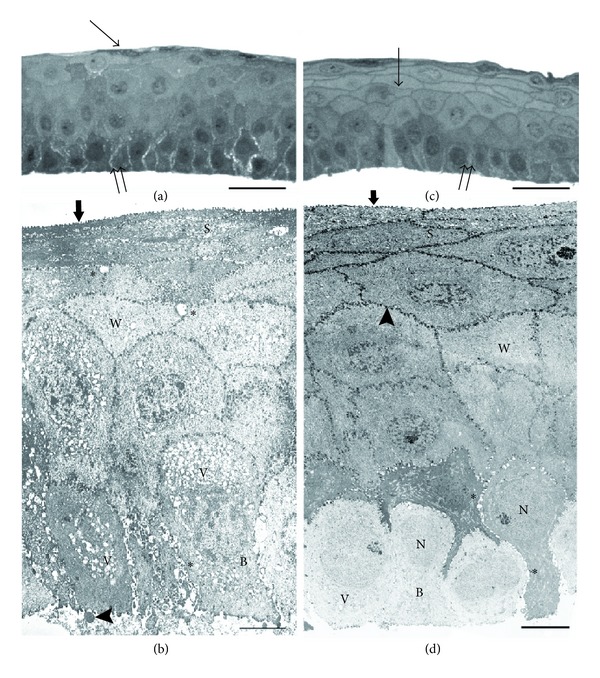
(a) Light micrograph from a semithin section of the corneal epithelium obtained with alcohol delamination in TUE. Superficial cells show variable shape and optical density (arrow), basal cells have hyperchromic nuclei and large intercellular spaces (double arrow). Scale bar: 25 *μ*m. (b) TEM micrograph of the corneal epithelium obtained with alcohol delamination in TUE. Superficial cells (S) show a reduced number of apical microfolds (arrow). In the wing cells (W) intracellular vesicles and slightly dilated intercellular spaces (∗) are evident. Basal cells (B) are irregular in their shape and size and show large intercellular spaces (∗) and a cytoplasm filled with vesicle (V). Many blebs (arrowhead), surrounded by granular material, are evident. Scale bar: 5 *μ*m. (c) Light micrograph from a semithin section of the corneal epithelium in TTE. Both superficial and wing cells show normal shape and well-evident intercellular borders (arrow); basal cells have polygonal shape and many small intracytoplasmatic vesicles (double arrow). Scale bar: 25 *μ*m. (d) TEM micrograph of the corneal epithelium in TTE. Superficial cells (S) show a normal flattened shape with well-evident apical microfolds (arrow). Wing cells (W) have uniform electron density and normal intercellular spaces (arrowhead). Basal cells (B) have variable electron density, polygonal shape, few cytoplasmatic vesicles (V), round nuclei (N), and small intercellular spaces (∗). Scale bar: 5 *μ*m.

**Figure 3:**
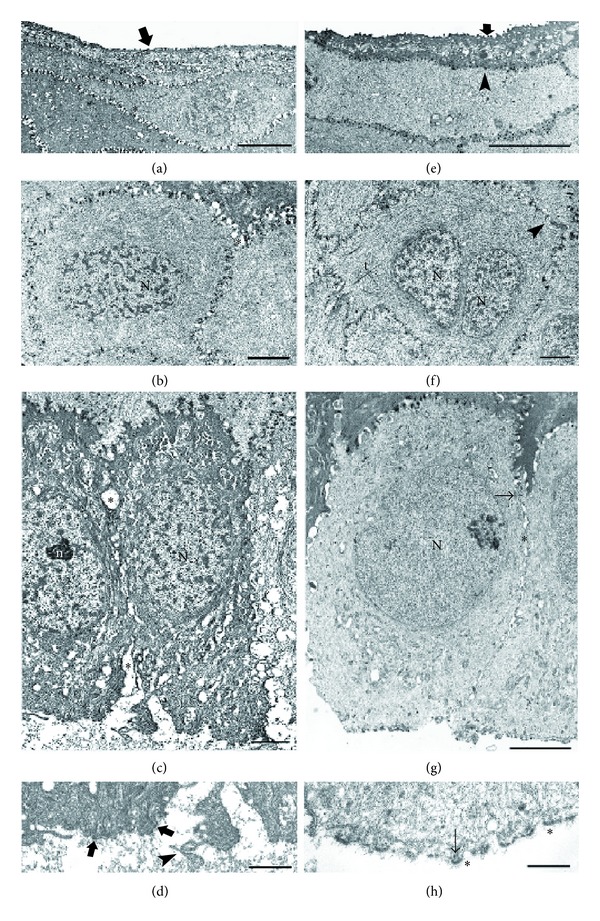
(a) TEM micrograph of superficial cells from the corneal epithelium obtained with alcohol delamination on TUE. The cells are flat and show short and irregular microfolds (arrow) and dilated intercellular spaces (∗). Scale bar: 5 *μ*m. (b) TEM micrograph of wing cells from the corneal epithelium obtained with alcohol delamination on TUE. The nucleus (N) and the cytoplasm have normal appearance; intercellular spaces are moderately dilated (∗). Scale bar: 2 *μ*m. (c) TEM micrograph of basal cells from the corneal epithelium obtained with alcohol delamination on TUE. The cells show elliptical nuclei (N) with evident nucleoli (n), dark and vacuolated cytoplasm, and dilated intercellular spaces (∗). Scale bar: 2 *μ*m. (d) TEM micrograph of the inferior pole of a basal cell from the corneal epithelium obtained with alcohol delamination on TUE. The cytoplasm is dense and few hemidesmosomes (arrows) are present. Note the presence of cellular blebs (arrowhead) and of an irregular granular material. Scale bar: 0.5 *μ*m. (e) TEM micrograph of superficial cells from the corneal epithelium of TTE. The cells are flat, show regular microfolds (arrow), and are well-preserved intercellular borders (arrowhead). Scale bar: 5 *μ*m. (f) TEM micrograph of wing cells from the corneal epithelium of TTE. Many tonofilaments (t) are evident around the apparently double nucleus (N_1_-N_2_); intercellular borders are normal, with well-evident desmosomes (arrowhead). Scale bar: 2 *μ*m. (g) TEM micrograph of basal cells from corneal epithelium in TTE. Basal cells have round euchromatic nucleus (N), clear cytoplasm with only few vesicles, small intercellular widenings (∗), and some desmosomes (arrow). Scale bar: 2 *μ*m. (h) TEM micrograph of the inferior pole of a basal cell from the corneal epithelium in TTE. The cytoplasm is clear and the hemidesmosomes (arrows) are numerous. ∗ = lamina lucida of the basement membrane. Scale bar: 0.5 *μ*m.

The corneal epithelium, obtained from TUE and observed in semithin sections at light microscopy, showed superficial cells with variable shape and optical density, round wing cells with not clearly evident intercellular borders, and basal cells with hyperchromic nuclei and wide intercellular spaces (Figure 2(a)). With TEM, corneal superficial cells were flat and showed few, short, irregular microfolds and dilated intercellular spaces (Figures 2(b) and 3(a)). Wing cells showed no apparent changes, whilst intercellular spaces were moderately dilated (Figures 2(b) and 3(b)). Basal cells had elliptical nuclei with evident nucleoli, dark and vacuolated cytoplasm, and dilated intercellular spaces (Figures 2(b) and 3(c)). Their inferior pole showed dense cytoplasm, few Hd, and some blebs surrounded by aggregates of granular material (Figures 2(b) and 3(d)).

In the corneal epithelium obtained from TTE, observed in semithin sections at light microscopy, superficial cells appeared flat with well-evident intercellular borders; wing cells were polygonal with well-evident intercellular borders; and basal cells had polygonal shape and evident intercellular spaces (Figure 2(c)). With TEM, corneal superficial cells were flat and showed regularly arranged microfolds and well-preserved intercellular borders (Figures 2(d) and 3(e)). Wing cells showed well-evident cytoskeleton and regular intercellular borders, glued by many De (Figure 2(d)). Occasionally, some wing cells, otherwise, of normal morphology, showed an apparently double nucleus (Figure 3(f)). Basal cells showed a clear cytoplasm with only few vesicles, a round, euchromatic nucleus, small intercellular widenings, and some desmosomes (Figures 2(d) and 3(g)). Their inferior pole showed clear cytoplasm and numerous Hd connected to the lamina lucida of the basement membrane (Figure 3(h)).

### 3.3. Morphometric Data

The results of the morphometric analysis within each group showed (Table 1) that, for basal cells, in TUE the number of De was 9.2 ± 1.1 between clear/clear cells, 5.7 ± 1 between clear/dark cells, and 4.2 ± 1 between dark/dark cells (clear/clear versus clear/dark *P* < 0.0001, clear/clear versus dark/dark *P* < 0.0001, and clear/dark versus dark/dark *P* < 0.0001). For Hd, a statistically significant difference was observed in their number when clear and dark basal cells were compared (21.9 ± 2.9 and 18.1 ± 1.5, resp., *P* < 0.0001). In TTE, the number of De was 9.4 ± 1.6 between clear/clear cells, 6.3 ± 0.8 between clear/dark cells, and 5.7 ± 1 between dark/dark cells (clear/clear versus clear/dark *P* < 0.0001, clear/clear versus dark/dark *P* < 0.0001 and clear/dark versus dark/dark *P* = 0.04). For Hd, a statistically significant difference was observed in their number when clear and dark basal cells were compared (24 ± 2.1 and 20.9 ± 2.4, resp., *P* < 0.0001).

Comparing the two groups (Table 1), a statistically significant higher epithelial thickness in TTE was demonstrated (57.7 ± 4.1 *μ*m in TTE and 55.4 ± 3.5 *μ*m in TUE, resp.; *P* < 0.0001). As the number of De, their number was statistically significantly higher in TTE between superficial cells (27.1 ± 3.1 in TTE and 20.1 ± 2 in TUE, resp.; *P* < 0.0001) and between dark basal cells (5.7 ± 1 in TTE and 4.2 ± 1 in TUE, resp.; *P* < 0.0001). As to the number of Hd present in the inferior pole of the basal cells, it was statistically significantly higher in TTE in both the clear (24 ± 2.1 in TTE and 21.9 ± 2.9 in TUE, resp., *P* < 0.0001) and the dark cells (20.9 ± 2.4 in TTE and 18.1 ± 1.5 in TUE, resp., *P* < 0.0001).

No statistically significant differences between both groups were found for the number of the cellular layers and for the number of De between wing cells, clear/clear basal cells, and clear/dark basal cells.

**Figure 4:**
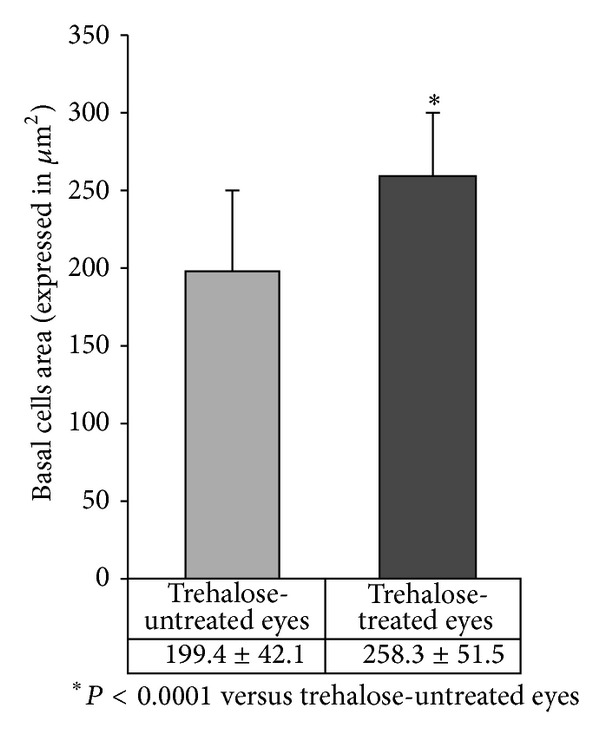
Morphometric data of the basal cells area in TUE and TTE expressed in *μ*m^2^. ∗ = *P* < 0.0001.

When the basal cells area was evaluated, a statistically significant difference was found between TUE and TTE (199.4 ± 42.1 *μ*m^2^ and 258.3 ± 51.5 *μ*m^2^, resp.; *P* < 0.0001) (Figure 4).

**Figure 5:**
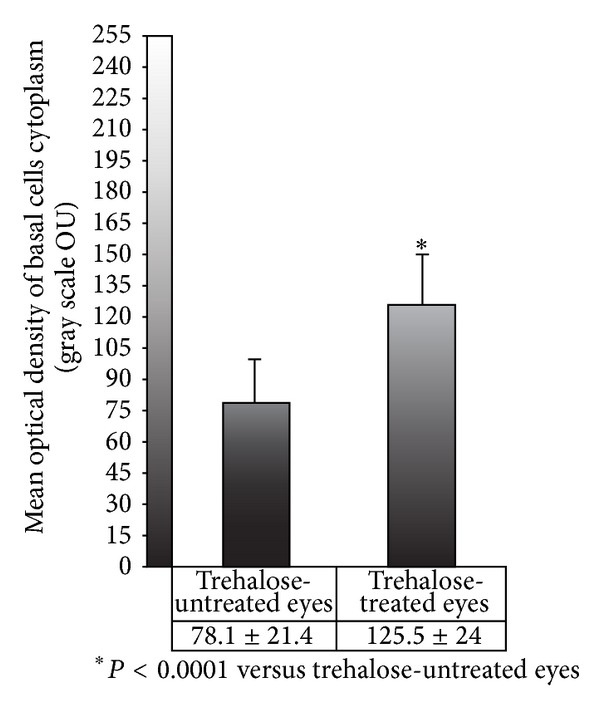
Morphometric data of the optical density of the basal cells cytoplasm in TUE and TTE expressed within the levels from 0 (black) to 255 (white) optical units (OU). ∗ = *P* < 0.0001.

Considering the optical density of the cytoplasm of the basal cells, a statistically significant difference was found between TUE and TTE (125.5 ± 24 OU and 78.1 ± 21.4, resp.; *P* < 0.0001) (Figure 5).

## 4. Discussion

Diluted ethanol is currently used in patients undergoing LASEK to reduce the adhesion of the corneal epithelium to Bowman's layer, in order to expose the stroma for the subsequent PRK treatment. The theoretical advantage of LASEK is derived from the repositioning of the epithelial flap over the laser-ablated corneal surface, so facilitating the corneal epithelial healing, reducing pain and inflammation, and decreasing the stromal haze [4].

However, adverse effects of alcohol on the corneal epithelial cells have been shown. In particular, the following changes were described: flattening of apical microvilli [3] and dark cytoplasm in the superficial cells [11], diffuse interruptions of the intercellular junctions with enlargement of the intercellular spaces [3, 8, 9], cellular edema [3], blebs of the cellular membrane [5, 8, 9], autophagic vacuoles [5], damages of Hd and of the basement membrane [4, 5], and coexistence of clear and dark cytoplasm in the basal cells [5, 8, 11]. Furthermore, the treatment with alcohol resulted in an increase of apoptotic basal cells, in both laboratory animals [2, 4] and humans [5].

Therefore, a treatment able to reduce the structural and ultrastructural changes induced by the exposition to diluted ethanol, characterized by the absence of side effects, can be considered useful in maintaining a healthier corneal epithelium, thus possibly improving its morphological and functional recovery after the surgical procedure.

Trehalose, a naturally occurring alpha-linked disaccharide formed by two molecules of glucose, was considered for its well-known protective effects in eukaryotic cells [15]. Trehalose, synthesized by many living organisms, but not mammals, showed many different functions. In anhydrobiosis, that is, the capability of surviving prolonged periods of dessication, a better resistance to dryness conditions was obtained by increasing the intracellular trehalose levels in animal cells [15]; in this way, proteins and membranes were protected from denaturation [20]. A reduced death by desiccation of human corneal epithelium in culture was obtained with the pretreatment of the cells with a 100 mM solution of trehalose [16]. Furthermore, trehalose might act as free radicals scavenger, reducing the oxidative damage of the cornea caused by UVB rays and suppressing the expression of proinflammatory cytokines [21]. Finally, trehalose based eye drops were found to be effective in the treatment of moderate to severe human dry eye [17], as it reduced the number of dead cells on the ocular surface through the suppression of apoptosis [18].

In the present study, trehalose dosage was chosen on the basis of previous experiences [16], which demonstrated the effect of the exposure to trehalose for 15′ on corneal epithelial cells maintained in culture. This procedure of administration was adopted, as it fitted appropriately the timing of the surgical procedures.

The positive action of trehalose on the corneal epithelium could be demonstrated by the reduction of the morphologic changes in TTE and by the evaluation of the morphometric data.

As to the morphological aspects, structural changes were evident in TUE. In fact, a reduced number and length of the apical microfolds of the superficial cells, a dark and vacuolated cytoplasm of the basal cells, a lower number of Hd, and a diffuse enlargement of the intercellular spaces were observed. On the contrary, in TTE superficial cells showed well represented microfolds and basal cells had clear cytoplasm with few vesicles and showed less evident intercellular spaces.

Basal cells with clear cytoplasm and euchromatic nucleus are considered the normal cells of the corneal epithelium [22]. Noxae acting on the cornea were shown to transform the structural and ultrastructural morphology of the basal cells, which become darker with more condensed chromatin and cytoplasm and wider intercellular spaces. These morphological aspects have been described either in experimental, such as induced vitamin A deficiency [23] or in pathological conditions, such as macular corneal dystrophy [24] and hereditary Thiel-Behnke corneal dystrophy [25], and were related to the concomitant deficiencies in Bowman's layer [24].

It is well known that, following ethanol treatment, the intracellular water is removed and replaced by alcohol itself, with consequent changes in the tertiary structure of proteins and cellular shrinkage [14]. On the contrary, when the corneal epithelium was pretreated with trehalose, this molecule, by substituting water molecules and forming hydrogen bonds, could stabilize the three-dimensional structure of the proteins [26].

The protective role of trehalose on the corneal epithelium was also supported by our morphometric results. In fact, in both corneal epithelial groups, the number of layers was superimposable and the epithelial thickness was within the normal values [22]. However, in TTE the epithelium was statistically significantly thicker than TUE. Furthermore, TTE showed statistically significantly higher values than TUE for basal cells area and cytoplasmatic density. It was thus possible to propose that the disaccharide could preserve the physiological morphology of the epithelial cells, in contrast with the darker, vacuolated appearance induced by the ethanol alone.

The morphometric analysis showed also that the number of De between adjacent superficial cells was statistically significantly higher in TTE. In addition, in both groups, De of adjacent basal cells were statistically significantly less numerous between dark cells, when compared to the junctions between clear cells or clear/dark cells. Even if no statistical data are currently available, as far as we know, on De distribution in the intact corneal epithelium, these data could indicate a better adhesion between cellular membranes in TTE and explain the morphological findings of wider intercellular spaces observed between dark cells.

As to the Hd found in the inferior pole of the basal cells, their mean number was calculated in 10 *μ*m of basal membrane, in order to obtain data from single basal cells. In fact, their ultrastructural aspect was uneven, being characterized by the coexistence of clear and dark cells. In this way, the analysis of a single cell could be performed and the values could be referred to as the specific morphological cell type. Clear cells in the TTE showed normal values of Hd, when compared to what reported in a previous work [27], whereas clear cells in TUE and dark cells in both groups showed statistically significantly lower values. In clear cells, Hd maintained their regular connection with the lamina lucida of the basement membrane, thus confirming that the cleavage plane of ethanol-induced corneal epithelial flaps was located between the lamina lucida and the lamina densa of the basement membrane [7]. On the contrary, in dark cells membrane-bound blebs of the basal pole and an extracellular granular-filamentous material, similar to that observed in patients with recurrent corneal erosions [12], were present, thus indicating an irregular cleavage of these cells.

## 5. Conclusions

The evidence reported in previous studies, indicating that trehalose is able to improve the corneal epithelial conditions in course of diseases such as dry eye, together with our findings, shows that it has a direct therapeutic role on the epithelial cells: therefore, its use could be advantageous in the treatment of patients with epithelial damage. However, further studies about the clinical outcome are needed to confirm the validity of its use.

## Figures and Tables

**Table 1:** Morphometric parameters of the corneal epithelium in trehalose-untreated eyes (TUE) and trehalose-treated eyes (TTE).

	Corneal epithelium		Desmosomes (number/10 *μ*m)					Hemidesmosomes (number/10 *μ*m)	
	Number of layers	Thickness (*μ*m)	Between superficial cells	Between wing cells	Between basal cells			Basal cells	
					Clear/clear	Clear/dark	Dark/dark	Clear cells	Dark cells
TUE	6.7 ± 0.5	55.4 ± 3.5	20.1 ± 2	26.5 ± 2.2	9.2 ± 1.1^†^	5.7 ± 1^†^	4.2 ± 1^†^	21.9 ± 2.9	18.1 ± 1.5^‡^
TTE	6.8 ± 0.7	57.7 ± 4.1∗	27.1 ± 3.1∗	24.4 ± 5.1	9.4 ± 1.6^††^	6.3 ± 0.8^*≠*^	5.7 ± 1∗	24 ± 2.1∗	20.9 ± 2.4^∗‡^

*P < 0.0001 versus TUE.

^†^ *P* < 0.0001 versus the other cell junctions within the TUE group.

^††^ *P* < 0.0001 versus the other cell junctions in TTE group.

^≠^ *P* = 0.04 versus dark/dark cell junctions in TTE group.

^‡^ *P* < 0.0001 versus clear cells of the same group.
